# Chloride Accumulators NKCC1 and AE2 in Mouse GnRH Neurons: Implications for GABA_A_ Mediated Excitation

**DOI:** 10.1371/journal.pone.0131076

**Published:** 2015-06-25

**Authors:** Carol Taylor-Burds, Paul Cheng, Susan Wray

**Affiliations:** Cellular and Developmental Neurobiology Section, NINDS/NIH, Bethesda, Maryland, United States of America; John Hopkins University School of Medicine, UNITED STATES

## Abstract

A developmental “switch” in chloride transporters occurs in most neurons resulting in GABA_A_ mediated hyperpolarization in the adult. However, several neuronal cell subtypes maintain primarily depolarizing responses to GABA_A_ receptor activation. Among this group are gonadotropin-releasing hormone-1 (GnRH) neurons, which control puberty and reproduction. NKCC1 is the primary chloride accumulator in neurons, expressed at high levels early in development and contributes to depolarization after GABA_A_ receptor activation. In contrast, KCC2 is the primary chloride extruder in neurons, expressed at high levels in the adult and contributes to hyperpolarization after GABA_A_ receptor activation. Anion exchangers (AEs) are also potential modulators of responses to GABA_A_ activation since they accumulate chloride and extrude bicarbonate. To evaluate the mechanism(s) underlying GABA_A_ mediated depolarization, GnRH neurons were analyzed for 1) expression of chloride transporters and AEs in embryonic, pre-pubertal, and adult mice 2) responses to GABA_A_ receptor activation in NKCC1^-/-^ mice and 3) function of AEs in these responses. At all ages, GnRH neurons were immunopositive for NKCC1 and AE2 but not KCC2 or AE3. Using explants, calcium imaging and gramicidin perforated patch clamp techniques we found that GnRH neurons from NKCC1^-/-^ mice retained relatively normal responses to the GABA_A_ agonist muscimol. However, acute pharmacological inhibition of NKCC1 with bumetanide eliminated the depolarization/calcium response to muscimol in 40% of GnRH neurons from WT mice. In the remaining GnRH neurons, HCO_3_^-^ mediated mechanisms accounted for the remaining calcium responses to muscimol. Collectively these data reveal mechanisms responsible for maintaining depolarizing GABA_A_ mediated transmission in GnRH neurons.

## Introduction

The GABA_A_ receptor is a Cl^-^ and, to a lesser extent, HCO_3_^-^ channel, which hyperpolarizes or depolarizes neurons depending on the ion driving force. Transporters and exchangers are critical in establishing, maintaining or changing these gradients. Over development many neurons undergo a switch in their response to GABA_A_ activation from depolarizing to hyperpolarizing as expression of Na+-K+-Cl—co-transporter-1 (NKCC1) decreases and expression of K+-Cl—cotransporter-2 (KCC2) increases (for reviews see [1, 2]). Depolarizing GABA_A_ responses in adult neurons correlate with a lack of KCC2 function in diseases [3]. However, it is now clear that interneurons in the hippocampus [4] and neurons in the hypothalamus [5–7] exhibit depolarizing responses to GABA_A_ activation in normal adult animals. In the hypothalamus these include magnocellular vasopressin neurons [5], neurons in the suprachiasmatic nucleus (6) as well as Gonadotropin-releasing hormone-1 (GnRH) neurons [7, 8]. GABA_A_ excitation in adult animals, likely reflects a different pattern of Cl^-^ transporter expression.

GnRH neurons within the forebrain are essential for vertebrate reproduction, with GnRH release into the portal blood system stimulating luteinizing hormone and follicle stimulating hormone from the pituitary [9]. Roughly ~70% of GnRH neurons maintain a depolarizing/excitatory response to GABA_A_ activation in the adult [8]. DeFazio et al., (2002) examined the transcript for NKCC1 and KCC2 in adult mouse GnRH neurons and identified only ~14% of cells positive for NKCC1, and less than 5% positive for KCC2. These data suggest that GnRH neurons never develop KCC2 and that NKCC1 expression and Cl^-^ accumulation decrease developmentally leading to a “shift” in Cl^-^ [10] rather than the classical “switch” that has been described in many KCC2 expressing CNS neurons [1, 2]. The lack of KCC2 expression in GnRH neurons is similar to that found in some peripheral neurons [10]. However, unlike the values reported for GnRH neurons [7], the KCC2 negative peripheral neurons maintained expression of NKCC1 throughout development [10]. Intracellular Cl^-^ levels can also be modulated by Cl^-^/HCO_3_^-^ exchangers including the Na^+^ dependent Cl^-^/HCO_3_^-^ exchangers (NDCBEs) and Na independent anion exchangers (AEs) [11]. Functional AEs have been shown to lead to chloride accumulation in peripheral neurons [12] and the transcripts of AE2 and AE3 have been localized to neurons [13–16], with AE3 appearing to contribute to Cl^-^ accumulation in several types of neurons [17–19]. Thus, to understand the primarily depolarizing responses of adult neurons to GABA_A_ activation, the developmental pattern and function of Cl^-^ transporters and AE2 and AE3 was examined in GnRH cells.

This report shows that GnRH cells are immunopositive for NKCC1 and AE2 at embryonic, pre-pubertal, and adult ages. In contrast, KCC2 and AE3 were not detected in GnRH neurons at any age. The role of NKCC1 in GABA_A_ mediated responses was evaluated using primary GnRH neurons maintained in explants generated from NKCC1^+/+^ and NKCC1^-/-^ mice. Using pharmacological and transgenic approaches we found that NKCC1 and AE2/HCO_3_^-^ dependent exchangers contributed to maintaining GABA_A_ mediated excitation in GnRH neurons.

## Material and Methods

### Animals

All procedures were approved by NINDS IACUC (ASP# 1221–15) and performed in accordance with NIH guidelines.

### Mice

NKCC1^+/-^ mice were a gift from Dr. G. Shull (University of Cincinnati College of Medicine) and genotyping was performed as previously described [20]. GnRH-GFP mice were a gift from Dr. D. Spergel [21]. NKCC1^+/-^ mice were crossed with GnRH-GFP^+/+^ mice to produce NKCC1^+/-^/GnRH-GFP^+/-^. These mice were mated to obtain NKCC1^+/+^/GnRH-GFP^+/-^ (“NKCC1^+/+”^) mice and NKCC1^-/-^/GnRH-GFP^+/-^ (“NKCC1^-/-“^) mice. Tissue was also taken from GnRH-GFP^+/+^ and from GnRH-GFP^+/-^ mice.

### In vivo

Time mated females were euthanized in a CO_2_ chamber followed by cervical dislocation. Embryos (E12.5-E14.5) were removed, immersed in 4% Formaldehyde/PBS (1–2 hrs), rinsed in PBS, cryoprotected (30% sucrose), embedded in Tissue Tek OCT and stored at -80°C until cutting. Pre-pubertal mice (postnatal day [PN] 9–12) were euthanized in a CO_2_ chamber, followed by cervical dislocation and brains removed. Adult mice (PN 45–80) were killed with an overdose of 200mg/kg ketamine and transcardially perfused with 1X PBS followed by 4% formaldehyde and brains removed. Pre-pubertal and adult brains were postfixed, cryoprotected and processed as above. Serial Sections (14–16 μm, 6 series/pre-pubertal and 8 series/adult) were cut on a Leica CM 3050S cryostat (Leica Biosystems) and maintained at -80°C until processing. N ≥ 3 for all stages, with both male and female pre-pubertal and adult mice examined, 2–3 series/animal.

### In vitro

Explants were generated as previously described [22, 23]. In brief, female mice were timed mated and euthanized at gestation day E11.5. Uteri were removed and transferred to cold PBS. The nasal placode was dissected out, and plated on thrombin + chicken plasma coated coverslips. GnRH neurons migrate out of the nasal pits during the first 5 days in vitro (div) and by 7div exhibited many characteristics of adult GnRH neurons *in vivo*. Explants were fed with defined, serum free media (SFM). A detailed description of the protocol and reagents can be found in [23]. Explants were used between 7-21div.

### PCR

Primers for AE2 and AE3 were designed from GenBank sequences (AE2: NM_009207.3; AE3: NM_009208.3) and screened using BLAST to ensure specificity: AE2: Forward: 5’-AACACCACCCAGATGTCACC, Reverse: 5’-GGCTCTGCCTCATTAGCATC; product size 229; AE3: Forward: 5’-TCTGGGTGGTCAAGTCAACG, Reverse: 5’- CCTAGGGTCTGT-GAATCGCC; product size 248. PCR for AE2 (S1 Fig) and AE3 (data not shown) was performed on single GnRH cell cDNAs (7div, n = 7; 28div, n = 7), 7div explants, and brain/testis (positive controls). Samples were prepared as described in [24], and the PCR was run for 40 cycles at 94°C for 30 sec, 62°C for 30 sec, and 94°C for 1-min. All cells were confirmed as GnRH positive. Amplified products were run on a 1.5% agarose gel. Specific bands of the predicted size were observed in control lanes (brain, testis), whereas no bands were detected in water. cDNAs from single cells were initially screened as described in [25–27].

### Antibodies

**Fig 1:**
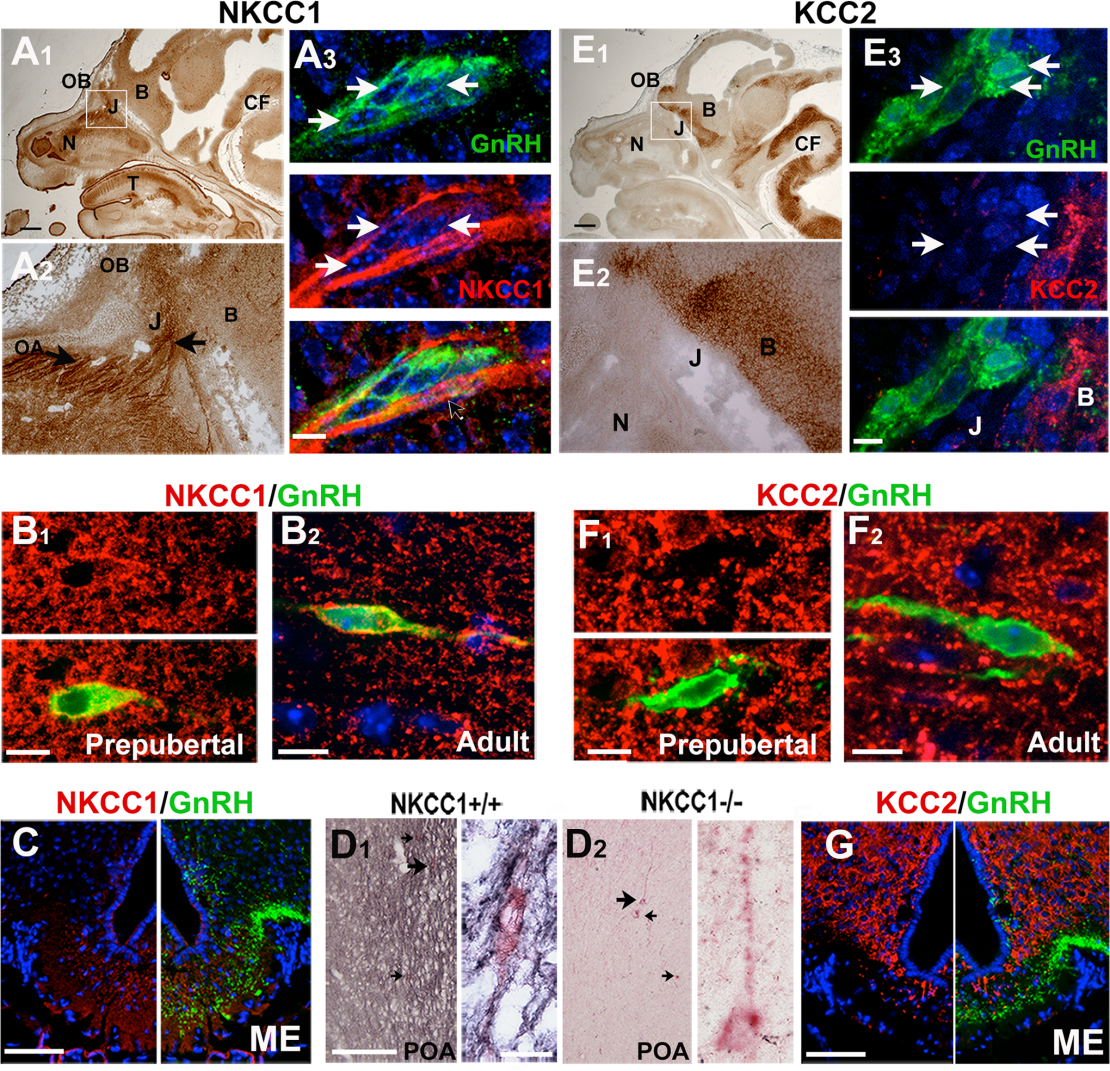
GnRH neurons are positive for NKCC1 and negative for KCC2 throughout development. A1-2) Robust NKCC1 staining (brown) was detected at E14.5 in the nasal area (N) including cells in the olfactory epithelium and cells and fibers (OA, black arrows) at the nasal forebrain junction (J), with many fibers seen entering the developing olfactory bulb (OB). A2 is higher magnification of boxed area in A1. A3) GnRH neurons (green, arrows) in nasal areas were NKCC1 positive (red), showing light labeling over their cell soma (white arrows). Labeling of GnRH processes was obscured by dense labeling of olfactory axon bundles (black arrow). B) NKCC1 (red) is co-localized in GnRH neurons (green) in pre-pubertal (B1) and adult (B2) mice. NKCC1 labeling was more robust in GnRH neurons and surrounding preoptic tissue at pre-pubertal ages compared to adult. However, in the adult, some GnRH neurons remained clearly positive for NKCC1 in the cell soma and proximal processes (B2). C) NKCC1 (red) was present in the median eminence (ME), but did not co-localized with GnRH fibers (green). D) Chromagen double-labeling amplification of NKCC1 signal with NiDAB (blue-black) in sections from WT (D1) and NKCC1^-/-^ (D2) mice demonstrates ubiquitous NKCC1 staining throughout the pre-optic area (POA) and staining of GnRH neurons (red), both cell soma and processes in WT mice (D1). E1-2) KCC2 staining (brown) was robust in the developing OB, ventral forebrain and areas around the cephalic flexure (CF). E2) Higher magnification of boxed area in E1 showing no KCC2 labeling in nasal areas (N) or at the nasal forebrain junction (J). E3) GnRH cells (green, arrows) at forebrain junction (J) are negative for KCC2 (red), while cells in the ventral forebrain (B) robustly stain for KCC2. F) GnRH cells (green) are KCC2 (red) negative in both pre-pubertal (F1) and adult (F2) mice, although robust KCC2 labeling is found throughout the brain. G) Although present in the median eminence (ME), KCC2 (red) did not co-localize with GnRH fibers (green). F) Double labeling was performed for GnRH neurons with anti-GFP and/or anti-cGnRH. Arrows indicate GnRH cells and large arrow indicate cells magnified on the right. Scale bars: A1 and B2 = 200 μM, E, F, and G = 100 μM; A3, B3, C and D = 10 μM. B = brain; T = tongue. N ≥ 3 at all stages.

Primary antibodies were rabbit polyclonal unless otherwise indicated: GnRH (SW-1, 1:1000) [28], Chicken anti-GnRH (affinity purified, 1:200; made by Aves Laboratory), monoclonal F1D3C5/GnRH (1:4000, gift from Dr. A. Karande, Dept of Biochemistry, Indian Institute of Science, Bangalore, India [29], Chicken anti-GFP (1:1000; Abcam), NKCC1 alphaCT (NKCC1, 1:1500) (Gift from Dr. J. Turner, NIDCR/NIH), NKCC1 TEFS-2 (NKCC1, 1:1500) (Gift from Dr. C. Lytle), KCC2 (1:600) (Millipore). AE2/AE1 (1:800, gift from Dr. S. Alper, Harvard Medical School), AE2 (1:500, Abcam), AE3 (1:600, Abcam), GFAP (1:400, Cell Signaling). NKCC1 antibodies were verified using fixed tissue of adult NKCC1^+/+^ and NKCC1^-/-^ mice (see Fig 1). For NKCC1, AE2 and AE3, antigen retrieval was performed prior to blocking (washed in PBS, 100% MetOH (5 min), rinsed in PBS, 1% SDS (5 min)). KCC2 staining was robust with or without antigen retrieval.

### Immunocytochemistry (ICC)

Single and double label chromagen ICC was performed on explants and tissue sections as previously described [23, 25] using standard avidin-biotin-horseradish peroxidase/3-diamino-benzidine (DAB), nickel-intensified DAB, or directly conjugated alkaline phosphatase secondary antibodies (Vector Laboratories, Inc., Burlingame, CA). Biotinylated secondary antibodies used were: donkey anti-rabbit (Vector Laboratories), donkey anti-mouse (Millipore Bioscience Research Reagents), and donkey anti-chicken (Jackson ImmunoResearch). For double labeling, antibodies from chicken (sections) and mouse (explants) were used for GnRH, while antibodies for NKCC1, KCC2, AE2 and AE3 were rabbit polyclonals. For each antibody, dilutions were established based on expression of each transporter in known tissue: NKCC1-chorid plexus; KCC2-hippcampus/cortex neurons; AE2-choroid plexus and ependymal cells; AE3-hippocampus (PN11). During the initial testing of antibody dilutions, negative controls lacking primary antibody incubation were included along with positive controls.

Double Immunofluorescence: Explants or sections were washed (PBS, ~40 min), blocked in 10% NHS with 0.3% Triton X-100 (1–2hr RT), rinsed (PBS) and placed in primary antibody 1–2 days (4°C). The following day tissue was washed (PBS), incubated in directly conjugated secondary antibody (1hr, RT), washed and steps repeated for second primary (different species) and secondary antibodies. Secondary antibodies used were: donkey anti-rabbit 555 or 568 (1:1000), donkey anti-mouse 555 (1:1000), goat anti-chicken 488 (1:1000) (all from Invitrogen). Sections were counterstained with 4,6-diamidino-2-phenylindole (DAPI; 1:3000/ PBS; Sigma-Aldrich), rinsed in PBS, and coverslipped with Fluoro Gel (Electron Microscopy Service). Confocal pictures were taken on a spinning disk confocal system (CSU10; Yokogawa) at 60X with a Nikon water immersion objective (NA 1.20) mounted on an Eclipse TSE200 microscope (Nikon) using an EMCCD ImageM digital camera (Hamamatsu) with I-Vision software (Biovision), or on a Nikon Eclipse E800 with a Retiga Exi camera using QCapture software (QImaging), as indicated. Images were further analyzed using NIH ImageJ software (W. Rasband, NIH, Bethesda, MD), and Figures assembled in Adobe Photoshop CS.

### Functional Assays

Calcium imaging and patch clamp recordings were performed on GnRH cells to delineate the transporter/exchangers underlying GABA_A_ mediated responses. All recordings were done using a chamber (Warner Instruments) with a gravity fed perfusion system (Warner Instruments) and Peristaltic pump (Spectra Spectra Hardware, Inc., Westmoreland City, PA). Cells were visualized with a 20X fluorescence objective (Nikon) on an inverted Microscope (Nikon TE2000). Perforated patch clamp recordings were done on a Multiclamp 700B amplifier (Molecular Devices) with a 1440A digidata (Molecular Devices) and analyzed with pClamp 10 (Molecular Devices). Electrodes were made from thick-walled borosilicate glass pulled to a resistance of 4–8 MΩ on a Flaming/Brown Micropipette Puller (Sutter Instrument). Tips were filled with filtered intracellular solution and backfilled with intracellular solution containing 10–15 μg/mL gramicidin (10 mg/mL DMSO stock solution made fresh). Gramicidin was added to fresh intracellular solution every 2–3 hr. Liquid junction potential and capacitance transients were canceled and access resistance (Ra) was monitored throughout recordings using Membrane Test in pClamp 10. Resting membrane potential (RMP) was checked in current clamp mode (I = 0), and remained stable once access resistance fell below ~300 MΩ. Reversal potentials for muscimol (E_MUS_) were obtained once access resistance had fallen to 50–80 MΩ. Spontaneous rupture of the patch was obvious from a fall in access resistance to below 15 MΩ, and dramatic increase in current. Perforation usually took 20–35 min. CO_2_/O_2_ bubbled ACSF was continuously run at ~0.8–1 ml/min. For E_MUS_ experiments, muscimol (MUS) was applied with a Picospritzer III (Parker Hannifin Corp). Electrodes used for drug application were the same as those used for cell recordings, and positioned ~30–40 μm from the cell soma, delivering a puff for 250 ms under 8–10 psi pressure. E_MUS_ estimations were done in voltage clamp with a holding potential of -60 mV, stepped (20mV) to +20 mV. Experiments measuring electrical activity and membrane depolarization were done in I = 0 mode once access resistance fell below 300 MΩ. Baseline activity and RMP was monitored for 2-min in CNQX and AP5 (10 μM each) before applying 1 μM MUS for 2-min. Some cells were re-tested in the presence of 1 μM TTX.

Calcium Imaging: Calcium Green-1 AM (Invitrogen) was used to measure relative changes in calcium activity using iVision 4.5 software (BioVision) as previously described, [24, 30]. A Lumencor Sola Light source was used with excitation 465–495 nm bandpass filter and an emission 40-nm bandpass centered on 535 nm. Briefly, explants were incubated in 13.5 μM Calcium Green-1 in a CO_2_ humidified incubator (37°C, 20 min), rinsed in ACSF and kept the incubator until use (30–40 min). Explants were moved to a recording chamber and perfused with CO_2_/O_2_ bubbled media ACSF (RT) for 30 min before recordings began. Images were acquired at 1 frame/sec. Recordings were terminated by 20 mM KCl to normalize calcium responses. Explants were fixed and stained for GnRH as described above. Regions of Interest were drawn over each GnRH cell and mean fluorescence (FL) intensity in each frame measured after subtracting background FL. GnRH-GFP positive cells had higher baseline FL intensity, but equivalent dynamic range for Ca^2+^. Calcium responses were measured using MATLAB software as described previously [31] and modified to measure peak amplitude minus baseline (mean of 5 frames before MUS or KCl was applied).

### Drugs and Solutions

Bumetanide (BUME: NKCC1 inhibitor), and Tetrodotoxin (TTX: voltage-gated sodium channel blocker) were obtained from Sigma. Muscimol (MUS: GABA_A_ receptor agonist), D-2-amino-5-phosphonovalerate (AP5: NMDA receptor antagonist) and 6-cyano-7-nitroquinoxaline-2,3-dione (CNQX: AMPA/kainate antagonist) were obtained from Tocris (Avonmouth, UK). All stock solutions (ACSF, ethanol, or dimethyl sulfoxide) were stored at -20°C. Solutions were prepared before each experiment by diluting stock solutions (1:1000 or 1:2000; vehicle controls were run if not previously tested). BUME was made fresh before each use. All other chemicals were purchased from Sigma. Calcium imaging and patch clamp experiments were performed in one of the following solutions: Bicarbonate/HEPES buffered ACSF (“ACSF”) or HEPES-only buffered ACSF (“HCO_3_^-^ free”). ACSF contained (in mM): 125 NaCl, 25 NaHCO_3_, 3 KCl, 1 MgCl_2_, 2.5 CaCl_2_, 5 HEPES (free acid), 20 glucose, and, pH 7.35–7.4 when bubbled with CO_2_/O_2_, osmolarity 310–320 mOsm/L. HCO_3_^-^ free ACSF contained: 150 NaCl, 3 KCl, 1 MgCl2, 2.5 CaCl_2_, 10 HEPES (free acid), 20 glucose, and, pH adjusted to 7.4 with NaOH 310–320 mOsm/L. Intracellular solution contained (in mM) 135 KCl, 10 K gluconate, with 1 MgCl_2_, 0.5 CaCl_2_, 10 HEPES free acid, and 5 EGTA, pH adjusted to 7.35 with KOH, osmolarity 290–300 mOsm/L. Solution osmolarity and glucose concentrations were based on the cell culture media, and has been described for electrophysiology previously [32].

Statistics: All statistics were performed using Prism Software V6 (GraphPad Software, Inc. La Jolla CA). Data were analyzed with one-way repeated measures or unpaired analysis of variance (ANOVA) followed by Bonferroni correction for multiple comparisons post-hoc tests as appropriate. Comparisons between groups of two were done using two-tailed unpaired or paired t-tests where appropriate. Significance was set at P≤0.05. n = number of cells, N = number of explants or animals. Each experimental group consisted of animals from at least two different litters. Data are presented as mean ± S.E.M.

## Results

### Developmental Expression of Chloride Transporters and Anion exchangers

**Fig 2:**
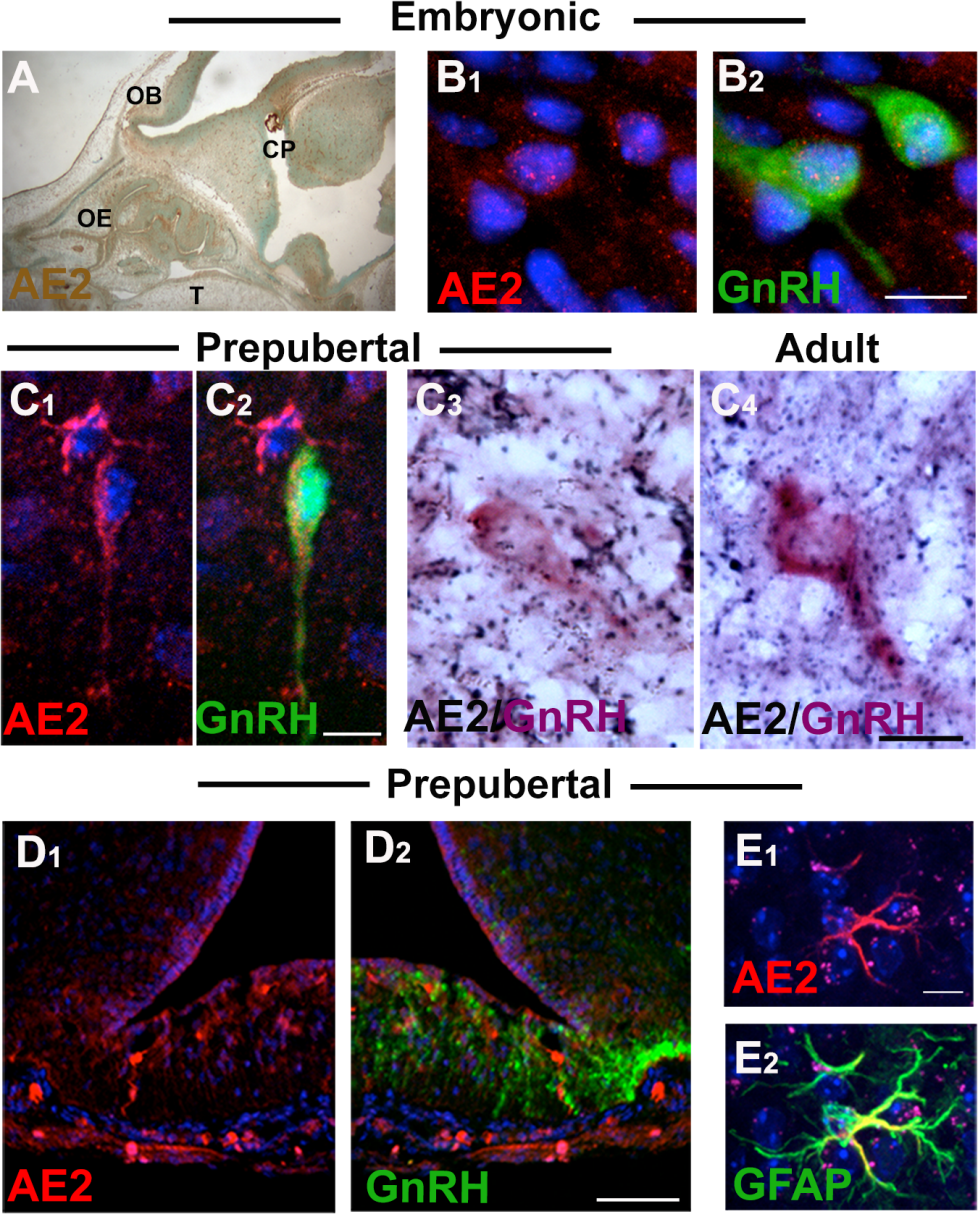
Anion exchanger 2 (AE2) is expressed in GnRH neurons throughout development. GnRH cells (green) in prenatal (A, B) and pre-pubertal mice (C1-2) expressed AE2 (red). C3-4) Chromagen intensified double-labeling for AE2 (NiDAB, black) and GFP (ABC-AP, purple) confirmed that GnRH neurons express AE2 in pre-pubertal (C3) and adult (C4) mice. D) No AE2 (red) expression was detected on GnRH fibers (green) in the median eminence, whereas astrocytes (GFAP, green) in neighboring regions were AE2 positive (E). Scale bars: B, C and E = 10 μM; D = 100 μM.

NKCC1 and KCC2 expression in GnRH neurons *in vivo* was examined at three different stages using chromagen immunocytochemistry and double-label immunofluorescence (Fig 1 and 2). Developmentally, GnRH neurons originate in the developing olfactory placode and migrate along olfactory axons/terminal nerve to the brain [33]. E14.5 was chosen for the early developmental time point because at this stage, GnRH cells are present in the nasal region, the nasal forebrain junction and within the brain (see Fig 1A_1_). Postnatally, GnRH cells are distributed in a rostral to caudal continuum from the olfactory bulbs to the caudal hypothalamus. GnRH cells throughout this continuum were examined for transporter and exchanger expression in pre-pubertal and adult tissues.

### Chloride Transporters

Staining of embryonic sections showed NKCC1 widely expressed throughout the embryo including the nasal area (Fig 1A_1–2_). Migrating GnRH cells were positive for NKCC1 (Fig 1A_3_). Examination of pre-pubertal (PN9-12) mice also showed GnRH cell bodies positive for NKCC1 (Fig 1B_1_). In adult mice, NKCC1 was still detected in GnRH cells (Fig 1B_2_). Comparing all stages, NKCC1 staining was most intense in pre-pubertal mice (Fig 1). The number of NKCC1 positive GnRH cells could not be quantified at E14.5 because of strong NKCC1 staining in olfactory axons (Fig 1A, black arrows). However, postnatal counts on double-labeled fluorescent material showed that the majority of GnRH cell somas were clearly NKCC1 positive in pre-pubertal mice (70%; n = 73/104, N = 2) and that there was a decrease in adults, (24%; n = 69/289; N = 7). Although NKCC1 was detected in the median eminence (ME), double labeled GnRH/NKCC1 processes were not found (Fig 1C). In adults, NKCC1 was detected throughout brain at very low levels. To maximize sensitivity and verify the ubiquitous presence of NKCC1 and its presence in GnRH neurons in adult mice, avidin-biotin nickel-enhanced DAB (Blue) was used on NKCC1^+/+^ and NKCC1^-/-^ tissue, double labeled for GnRH (Red; Fig 1D_1_ and 1D_2_ respectively). Comparison of sections from the two genotypes confirmed antibody specificity and confirmed NKCC1 was present throughout brain, including in the preoptic area that contains a large population of GnRH cells. Using this technique, NKCC1 was detected on proximal processes and cell bodies of most GnRH cells (Fig 1D high magnification). These data indicate that in adults, although the level of expression was lower, the number of NKCC1 positive GnRH cells was comparable to that found pre-pubertally.

In contrast to NKCC1 staining, KCC2 was absent from GnRH neurons at all ages. Migrating GnRH cells were negative for KCC2, which was not expressed in nasal areas though already present in brain regions [Fig 1E_1–3_]. Examination of pre-pubertal mice also showed GnRH cell bodies negative for KCC2 (Fig 1F_1_). In adult mice, no KCC2 positive GnRH neurons were found, even though robust KCC2 was detected in brains regions containing GnRH neurons (Fig 1F_2_). In fact, due to the abundance of KCC2 in postnatal tissues, co-localization required fluorescence labeling. Examination of GnRH-GFP mice (N = 4 male, 3 female) showed no GnRH neurons positive for KCC2. In cells where KCC2 fibers were in close proximity to GnRH, 20 cells were re-evaluated at 60X on a confocal microscope and analyzed in ImageJ. In every instance, GnRH cells were negative for KCC2 staining although KCC2 positive fibers often crossed over GnRH cell processes (Fig 1F_2_). Examination of GnRH processes in the OVLT (not shown) and ME were also clearly negative for KCC2 (Fig 1G).

### Anion Exchangers

Analysis of microarray data previously generated in our lab [34, 35] showed GnRH neurons had high levels of AE2 transcripts compared to AE3 (S1 Fig). Immunocytochemistry for AE2 and AE3 in GnRH neurons was performed *in vivo*. AE3 was not detected in GnRH neurons (N = 2 pre-pubertal, N = 2 adult), although it was expressed in non-GnRH fibers in the median eminence (S2 Fig). In contrast, two different antibodies confirmed the presence of AE2 in some, but not all, GnRH neurons in embryonic, pre-pubertal and adult mice (N = 4 pre-pubertal, N = 5 adult). AE2 positive GnRH cells were quantified after NiDAB intensification in pre-pubertal (N = 2), and adult (N = 3) mice (Fig 2 C3 and 2C4). No difference between ages was observed, so counts were combined. 55±1.3% of GnRH neurons showed AE2 immunoreactivity, 31±3% were negative, and 14±3% were not categorized due to the close proximity of AE2 positive fibers. AE2 was also present in the median eminence but did not appear to overlap with GnRH fibers although it was clearly detected in glial cells (GFAP+, Fig 2). Taken together these data indicate that GnRH neurons have the ability to use both NKCC1 and AE2 to regulate [Cl^-^]_i_.

### Functional Analysis

**Fig 3:**
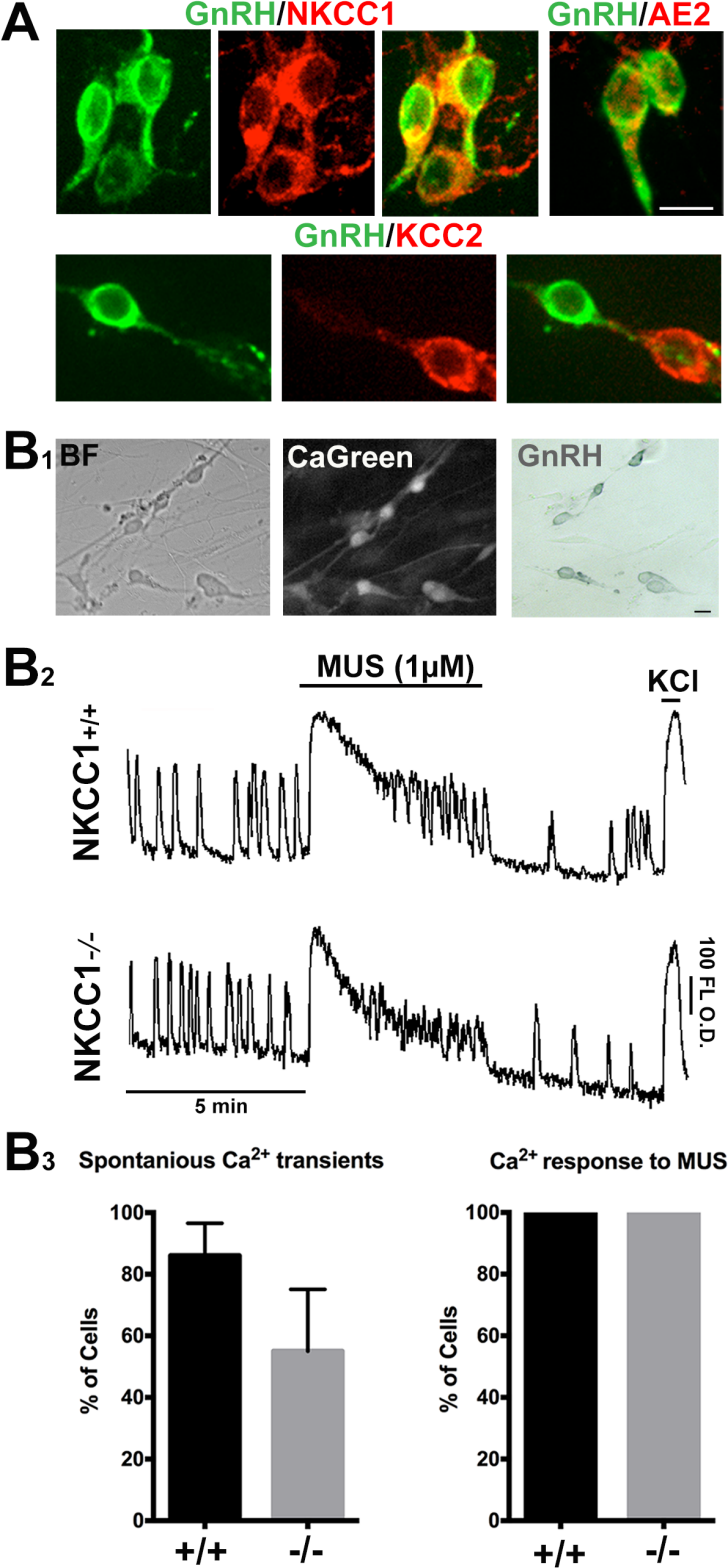
GnRH neurons retain depolarization to MUS in the absence of NKCC1. A) As in vivo, GnRH cells (green) maintained in explants express NKCC1 (red), and AE2 (red). GnRH neurons were negative for KCC2, although a few non-GnRH neurons expressed KCC2 (lower panel, GnRH neuron fiber near KCC2 positive cell). B1) Example of GnRH neurons used for calcium imaging: bright field (BF), after loading with the calcium indicator (CaGreen), and after fixing and staining (GnRH). (B2) Examples of calcium imaging traces from NKCC1^+/+^ and NKCC1^-/-^ GnRH neurons showed spontaneous activity in both genotypes (“Spontaneous Ca^2+^ transients”, B3). All GnRH neurons in both NKCC1^+/+^ (+/+) and NKCC1^-/-^ (-/-) explants had calcium responses to MUS (“Ca^2+^ responses to MUS”, B3). Error bars = S.E.M. O.D. = optical density. Scale bars, A and B1 = 10 μM.

To determine if NKCC1 and/or AE2 was critical for the depolarizing response to GABA_A_ activation, experiments were performed using intact primary GnRH neurons maintained in explants. The properties and receptors of GnRH cells in this model system mimic GnRH cells *in vivo* or slice preparations, and large numbers of GnRH cells are accessible for analysis [31]. Immunocytochemical analysis revealed that GnRH cells in 1–3 week old explants from WT mice, like GnRH neurons *in vivo*, expressed NKCC1 but not KCC2 (Fig 3A; N>4 for each group: 7–10, 14–17, and 21–24 div). A few GnRH negative/KCC2 positive cells were detected in most explants, however unlike GnRH cells, they did not migrate into the periphery. Their scarcity, and location in the tissue mass precluded them from analysis in imaging and recording experiments (Fig 3A). PCR on single cell cDNAs from GnRH neurons confirmed that transcripts for AE2 (S1 Fig) and AE3 (data not shown) were present in the majority of GnRH cells (7 div: AE2: 6/7, AE3: 5/7; and 28div: AE2: 4/7 cells, AE3: 4/8). However, protein expression of AE3 was not detected, while, similar to that found *in vivo*, ICC confirmed AE2 in some, but not all, GnRH neurons in explants (Fig 3A).

Calcium transients have been shown to correlate with electrical activity in GnRH cells in adult slices and explants [36–38]. Cl^-^ efflux with GABA_A_ activation leads to membrane depolarization and opening of Ca^2+^ channels. Thus, to evaluate the significance of NKCC1 in GnRH neurons, explants were generated from NKCC1^+/+^ and NKCC1^-/-^ mice and calcium imaging performed to monitor GnRH activity, and response to the GABA_A_ agonist MUS (Fig 3B). The majority of GnRH neurons under normal conditions are spontaneously active [38]. Calcium activity was monitored for 15 min in ACSF. In explants generated from NKCC1^+/+^ embryos, calcium transients were present in 86±6% of GnRH neurons (n = 119/137, N = 3). GnRH cells in explants generated from NKCC1^-/-^ embryos had slightly lower numbers, but not significantly different (p = .07), with 53±12% of GnRH cells exhibiting spontaneous calcium transient activity (n = 73/137, N = 3; Fig 3B). MUS (1 μM) was applied for 5 min in the presence of the ionotropic glutamate receptors antagonists (CNQX/AP5; 10 μM each) after establishing a baseline for 5 min (Fig 3B). MUS elicited large sustained calcium responses in the all of the cells, independent of genotype (NKCC1^+/+^: n = 137, N = 3; NKCC1^-/-^: n = 137, N = 3; Fig 3B). These data indicate that GnRH neurons lacking NKCC1^-/-^ were depolarized by GABA_A_ activation.

**Fig 4:**
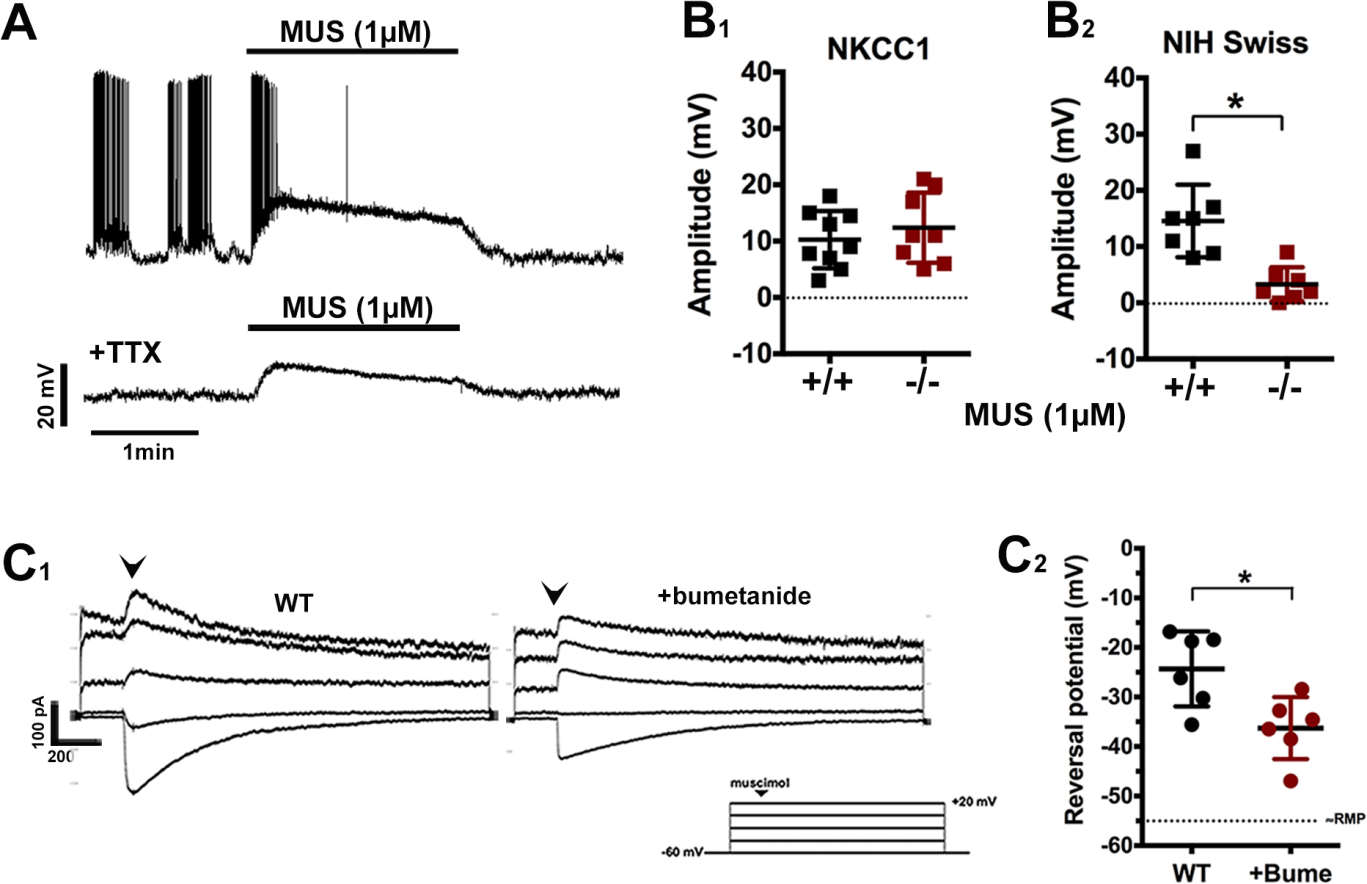
GABA_A_ activation elicits similar response in GnRH neurons from NKCC1^-/-^ explants but is reduced by BUME. A) Example of a gramicidin perforated patch clamp recording used to measure response to MUS. Trace from NKCC1^-/-^ GnRH cell shows MUS depolarization is present before and after TTX. B) Peak amplitudes of MUS responses in GnRH neurons are similar in NKCC1^+/+^ (+/+, black dots) and NKCC1^-/-^ (-/-, red dots) (B1) In contrast, BUME treatment significantly reduces the response to MUS in GnRH neurons in WT mice (B2). C) Reversal potential for GABA in GnRH neurons was evaluated by acute application of MUS (100 μM, arrowhead). All recordings were performed with CNQX/AP5 (10 μM each) and TTX (1 μM). Examples of recordings from GnRH neurons in untreated (WT) and bumetanide treated (+BUME) groups, and the voltage protocol used (C1). Reversal potentials were more negative when NKCC1 was inhibited by BUME (20 μM, red dots) than in control (WT, black dots) cells (*p<0.05, error bars = SEM) (C2).

Gramicidin perforated patch recording allow electrical access to the cell without disrupting intracellular Cl^-^ or signaling transduction mechanisms [39]. Thus, this technique was used to further evaluate the MUS response in GnRH neurons [39]. Recordings were carried out in current clamp I = 0 mode. CNQX/AP5 (10 μM each) was present throughout the recording. Resting membrane potential (RMP) was not different between GnRH neurons ± NKCC1 (NKCC1^+/+^: -62.33±2.21 mV, n = 9, N = 5; NKCC1^-/-^: -60.6±2.7 mV, n = 8, N = 4; unpaired t-test, p = 0.63). Peak depolarization by 1 μM MUS was also similar (NKCC1^+/+^: 10.3 ± 1.7 mV, n = 9; NKCC1^-/-^: 12.38±2.2 mV, n = 8, respectively, unpaired t-test, p = 0.45; Fig 4B_1_). The response to MUS was not significantly changed when re-tested in the presences of 1 μM TTX (example shown in Fig 4A; n = 5 cells, 3/NKCC1^+/+^ and 2 NKCC1^-/-^, Mean difference = -2.6±2.7 mV, paired t-test, p = 0.4).

Previous research has shown that tissue from NKCC1^-/-^ mice show compensatory changes for the loss of NKCC1 [40, 41]. Therefore we measured the MUS response in NIH Swiss WT mice (“WT”), either with or without the NKCC1 inhibitor Bumetanide (BUME). RMP was similar between GnRH neuronal responses in the absence (-61±2.7 mV, n = 7, N = 4) or presence of BUME (20 μM, bath applied for ≥ 20 min; -62±1.8 mV, n = 7, N = 5; unpaired t-test, p = 0.7). Unlike GnRH neurons in which NKCC1 had been genetically deleted, NKCC1^-/-^ mice, BUME treatment significantly reduced the peak depolarization caused by MUS (14.5±2.4 mV, vs 3.3±1.2 mV, p = 0.001; Fig 4B_2_). To confirm that BUME treatment reduced intracellular Cl^-^, the reversal potential of MUS was tested in the presence of CNQX/AP5 (10 μM each) and TTX (1 μM; to block action potential dependent mechanisms). Cells were voltage clamped at -60 mV, and stepped (20 mV) to +20 mV, with a brief (250 ms) application of MUS (100 μM to maximize current amplitude). E_MUS_ was ~11 mV more positive in the untreated WT group (-25.3±3.1 mV, n = 6/N = 5) than cells treated with BUME (-36.3±2.6 mV n = 6/N = 5, p = 0.01; Fig 4C). The competitive GABA_A_ antagonist PIC (100 μM) completely blocked the response to MUS in 4/4 cells tested (not shown). The patch was ruptured at the end of recording in one control cell and E_MUS_ retested. Whole cell reversal potential was -1.9 mV (not shown), close to the expected value calculated according to Nernst for E_Cl-_ (0.9 mV with: [Cl^-^]i = 133 mM; [Cl^-^]o = 128 mM at 22°C). These experiments indicate that acutely blocking NKCC1 significantly reduces, but does not eliminate the depolarization caused by GABA_A_ stimulation.

**Fig 5:**
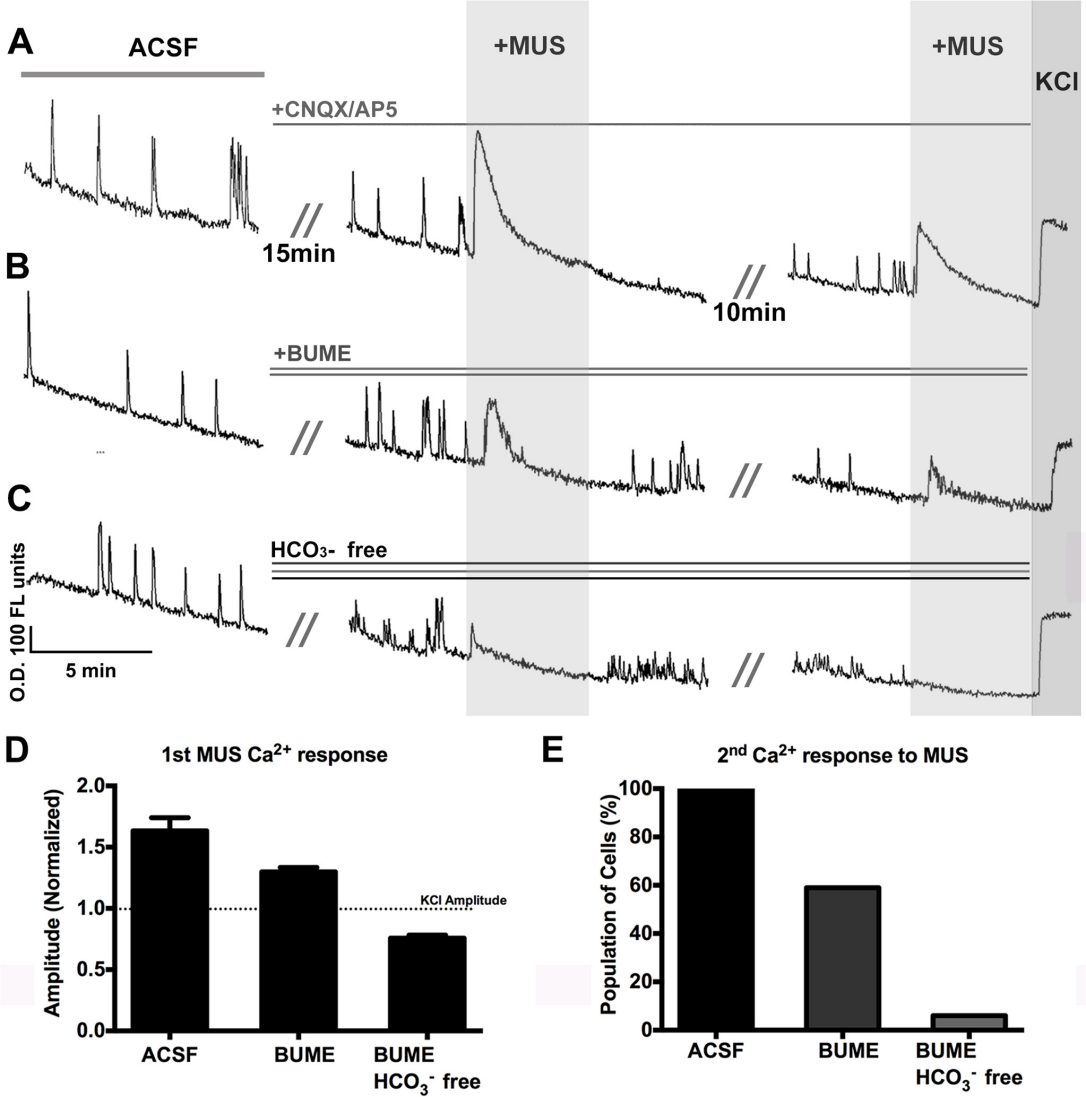
Inhibition of NKCC1 and removal of HCO3^-^ attenuates response to MUS. Recording periods consisted of: 1) baseline recording in ACSF, 2) application of CNQX/AP5 (10 μM each) followed by MUS (10 μM), and 3) second exposure to MUS (10 μM), followed by KCl (20mM). Groups compared were: A) Control B) BUME (NKCC1 inhibited), and C) HCO_**3**_^-^ free ACSF with BUME. Due to general fluorescent rundown in ACSF, all responses were normalized to KCl stimulus at the end, and amplitude comparisons were made. D) Amplitude of the 1st calcium responses to MUS. E) Percentage of cells retaining a calcium response to the second application of MUS. O.D. = optical density.

BUME blocks Cl^-^ accumulation via NKCC1, but does not eliminate Cl^-^ from the cell. Therefore, we attempted to rundown the Cl^-^ gradient by using a two-stimulus MUS protocol in explants from WT embryos, and monitored the calcium response elicited. This would allow us to determine if A) the remaining depolarization seen in BUME treated cells was due to another chloride accumulating mechanism, or B) it was due to residual Cl^-^ that was not depleted with NKCC1 inhibition. Cl^-^/ HCO_3_^-^ exchange from AEs has been reported to contribute to Cl^-^ accumulation in hippocampal neurons [18] and motoneurons [19]. Although no specific AE2 antagonist/blockers exist, removal of HCO_3_^-^ from the bath can be used to inhibit AE function since they are dependent on HCO_3_^-^ exchange with Cl^-^ [19, 42]. We tested the ability of GnRH neurons to re-accumulate Cl^-^ under three different conditions using a two-stimulus protocol. In the first control/untreated condition, NKCC1 and HCO_3_^-^ dependent mechanisms were not altered, allowing normal re-accumulation of Cl^-^ levels to be measured. In the second condition, NKCC1 function was blocked with BUME. In the third condition, both NKCC1 and AE function were eliminated using a combination of BUME in “HCO_3_^-^ free” ACSF (see Methods) rather than HCO_3_^-^/CO_2_ buffered ACSF. The protocol used is illustrated by example traces in Fig 5A–5C. To evaluate the relative amplitude of Ca^2+^ response in the three conditions, Ca^2+^ response amplitudes were normalized to the 20 mM KCl response, which was applied for 1 min at the end of the recording. This normalization also controlled for the gradual rundown in fluorescence intensity observed during Ca^2+^ imaging recordings (Fig 5A–5C). Amplitudes of the first Ca^2+^ response to MUS application (Fig 5D) were significantly different between the 3 groups [One way ANOVA, F(2, 389) = 57.03, p<0.0001]. Post-hoc analysis (Bonferroni's Multiple Comparison Test) showed that the response in BUME (Mean normalized amplitude optical density (O.D.) = 1.3±0.11) was significantly lower (p<0.0001) than the Control group (Mean normalized amplitude O.D. = 1.63±0.11), as was the difference between BUME and BUME/HCO_3_^-^ free groups (Mean normalized amplitude O.D = 0.76±0.03, p<0.0001). The second application of MUS produced either an attenuated Ca^2+^ response or no Ca^2+^ response (including loss of Ca^2+^ transients), dependent on the condition. All cells in the control group (n = 95, N = 3, Fig 5E) retained a Ca^2+^ response to the second application of MUS. In contrast, when BUME was present only 60% (n = 101/169, N = 4) of cells retained a Ca^2+^ response to the second application of MUS. When both NKCC1 and HCO_3_^-^ dependent mechanisms were blocked (BUME/HCO_3_^-^ free), only 5% of cells retained any Ca^2+^ response or Ca^2+^ activity (n = 6/128; N = 3). Calcium transients, and a large Ca^2+^ response to KCl were retained in cells from the HCO_3_^-^ free ACSF group indicating that inactivation of voltage gated Ca^2+^ channels did not account for the altered responses observed in the HCO_3_^-^ free solution. However, gramicidin perforated patch clamp recordings were performed in age-matched explants of BUME treated, or HCO_3_^-^ free/BUME ACSF. All cells were spontaneously active. RMP was slightly, but not significantly higher in HCO_3_^-^ free/BUME (n = 11, N = 7) than in ACSF/BUME (n = 8, N = 4) GnRH neurons (Mean = -52±1.3 mV, versus -56±1.1 mV respectively, p = 0.07, unpaired t-test). Although absence of HCO_3_^-^ may have a slight effect on the RMP, recorded values are still within the normal range reported for GnRH neurons both in our model system and *in vivo*, and do not account for the complete loss of the calcium response seen in the HCO_3_^-^ free/BUME ACSF group. These results demonstrate that, with respect to Cl^-^ accumulation, one group of GnRH neurons is dependent on NKCC1 alone (cells that were not excited by 10 μM MUS in BUME) while another group of GnRH neurons utilizes both NKCC1 and HCO_3_^-^ dependent mechanisms.

## Discussion

This report reveals mechanisms by which adult neurons can maintain depolarization after GABA_A_ receptor activation, and clarifies the mechanisms responsible for the mixed responses produced by GABA_A_ activation reported in GnRH neurons [8]. AE2 expression in the CNS has been largely overlooked, although it has been detected in neurons of the human brain [43], as well as in choroid plexus cells [44] and subventricular zone neuronal progenitor cells of the mouse [17]. We show that NKCC1 and AE2 are present in GnRH neurons throughout development (embryonic, pre-pubertal and adult) and that HCO_3_^-^ dependent processes can play a role in how GnRH neurons respond to GABA_A_ activation. Thus, our data support a model where the net effect of GABA_A_ activity in GnRH neurons becomes modulatory in adults; reduced expression/function of NKCC1 and AE2 leads to less Cl^-^ accumulation, and greater dependence on the state of the neuron to determine if GABA is excitatory or inhibitory.

Many CNS neurons switch from high to low intracellular Cl^-^ during development, which parallels a change in the expression of NKCC1 to KCC2 [45–48] and responses to GABA_A_ activation. KCC2 drives intracellular Cl^-^ well below the RMP via the K^+^ gradient, ensuring that GABA_A_ receptor activation will cause Cl^-^ influx/hyperpolarization. However some CNS neurons retain a primarily depolarizing or excitatory GABA_A_ response in adult animals [5, 6]. GnRH neurons were once thought to undergo a post-puberty switch to hyperpolarizing responses to GABA_A_ activation [49]. However, a series of studies revealed 60–80% of GnRH cells show excitatory/depolarizing responses to GABA_A_ [7, 50–55], although hyperpolarizing responses have still been reported [56]. Hyperpolarizing responses in adult GnRH neurons have been attributed to several possible methodical differences, including the absence of ionotropic glutamate antagonists [49] as well as the concentration and duration of GABA used [7]. This topic has been extensively discussed in a review [8]. However, the possible contributions of AE2 mechanisms were unknown at that time.

To better understand the mechanisms involved in Cl^-^ regulation, and therefore GABA_A_ activity, we examined chloride transporters and AEs in GnRH neurons over three different developmental time points, embryonic, early postnatal, and adult. In agreement with previous reports [7, 57] transcripts for NKCC1 and KCC2 were detected. In addition, AE2 and AE3 transcripts were also found in GnRH neurons. However using immunocytochemical approaches, only NKCC1 and AE2 were detected in GnRH neurons, at all developmental ages examined, and no sex differences were observed. To evaluate dependence on NKCC1 function for depolarizing responses to GABA_A_ activation, NKCC1^+/+^ and NKCC1^-/-^ mice were used. Depolarization from GABA_A_ mediated Cl^-^ efflux leads to Ca^2+^ influx via voltage gated Ca^2+^ channels [53, 58]. Therefore Ca^2+^ imaging as well as gramicidin perforated patch clamp techniques were employed. To our surprise the Ca^2+^ response and depolarization caused by MUS was similar in GnRH neurons from NKCC1^+/+^ and NKCC1^-/-^ mice.

To determine if NKCC1 was not functional, or if compensation in the knockout was occurring, the response to MUS was tested in the presence of the NKCC1 inhibitor bumetanide in GnRH cells from WT mice. Acute bumetanide treatment significantly reduced, but did not eliminate the depolarization by MUS, indicating compensation was occurring in the NKCC1^-/-^ animal. Since AE2 was present in many GnRH neurons, the contribution of HCO_3_^-^ -dependent mechanisms as well as NKCC1 function was tested on the Ca^2+^ response to MUS in WT mice. These experiments demonstrated that ~60% of the cells were able to re-accumulate enough Cl^-^ to retain Ca^2+^ responses to MUS when NKCC1 was inhibited, but not when HCO_3_^-^ was also depleted from the bath. Notably, the reversal potentials for E_MUS_ reported here, both with and without BUME, are ~10 mV more depolarized than those previously reported [7]. This may reflect differences in either the type of preparation used or the maturity state of the GnRH cells. Although we can not rule out differences in the preparation alter E_GABA_, the second possibility is consistent with our hypothesis that an immature state (pre-pubertal/7-28div) has higher Cl^-^ concentrations due to greater expression and perhaps activity of NKCC1 and AE2, which is down regulated/reduced in the adult.

Certainly one must use caution when interpreting results from global knockout animals where many changes (in addition to the loss of the target protein) may occur. The NKCC1^-/-^ mouse is significantly impaired, being deaf, having postural deficits, motor impairment, and spermogenesis failure, among other things [20, 59, 60]. NKCC1^-/-^ male mice are infertile while female mice can become pregnant, although not easily [60]. In NKCC1^-/-^ mice, many types of neurons show impaired Cl^-^ dependent responses including olfactory sensory neurons, [61], trigeminal ganglion neurons [62], and dorsal root ganglion neurons [59]. In the early postnatal CA3 region of the hippocampus, NKCC1^-/-^ mice have greatly reduced GABA-dependent giant depolarizing potentials [18, 40]. In contrast, GnRH neurons in explants from NKCC1^-/-^ mice showed relatively normal responses to the MUS. AE2 and/or other HCO_3_^-^ dependent mechanisms contribute to Cl^-^ regulation, and thus could be compensating for the loss of NKCC1 in GnRH neurons. Consistent with this hypothesis, Haering et al. recently reported increases in transcript for three Cl^-^ transporters including AE2 (Slc4a2), in the olfactory epithelium of NKCC1^-/-^ mice [41].

Cl^-^/HCO_3_^-^ exchangers appear to facilitate Cl^-^ accumulation in several types of neurons [16–19]. AE1-3 are electroneutral, exchanging intracellular HCO_3_^-^ for extracellular Cl^-^, thereby reducing intracellular pH and increasing intracellular Cl^-^. AE2 transport activity is modified by internal and external changes in pH, and varies between the isoforms [11]. The exchanger AE3 is most abundantly expressed in the CNS, loss of which protects against induced seizures [63]. AE3 protein was not detected in GnRH neurons or processes, although it was robustly expressed in the median eminence. In contrast, some GnRH neurons co-expressed AE2. Although AE2 is the most widely expressed Cl^-^/HCO_3_^-^ exchanger, it was found to be relatively low within the brain, except within choroid plexus cells [11, 44]. In the present study, immunostaining for AE2 required amplification, indicating low expression levels. However, using this technique clear AE2 labeling was detected in many brain regions, often in fibers. In addition, GFAP positive glia, primarily around ventricles and in the ME were strongly labeled by AE2. AE2 and AE3 are also expressed in the olfactory epithelium and may contribute to olfactory sensory neuron Cl^-^ accumulation [16]. GnRH neurons arise in the olfactory placode [64, 65]. Thus, the expression of AE2 in GnRH neurons may be due, in part, to their origin. Animals missing all five splice variants of AE2 die before weaning, in part due to GI tract problems [66], while knockout of only the AE2a and AE2b_1/2_ isoforms show male infertility due to spermiogenesis defects [67]. Unfortunately, the absence of specific inhibitors to the anion exchanger families makes precise determination of the AE function and contribution to cellular physiology extremely limited at this time.

In summary, we show that NKCC1 and AE2 are present and functional in GnRH neurons, whereas KCC2 and AE3 are not. AE2 is expressed in most, but not all GnRH neurons, and the protein appears to be lowest in adult animals. In contrast, NKCC1 is ubiquitous, although protein expression also appears to be reduced in the adult. Unlike many neurons where development leads to KCC2 expression and a Cl^-^ “switch”, our data are consistent with neurons that undergo a developmental “shift” in Cl^-^ as NKCC1 and AE2 expression decreases in adulthood. Thus GnRH neurons undergo a developmental reduction in Cl^-^ leading to lower, but still primarily depolarizing, GABA_A_ mediated transmission.

## Supporting Information

S1 Fig AE2 and AE3 transcripts are present in GnRH neurons. Microarray data generated in our lab (Kramer and Wray 2000) shows Mean mRNA transcript levels for 9 pooled single cell cDNAs in migrating (4 div) and post-migrating (10 div) GnRH neurons for AE2 (Slc4a2) and AE3 (Slc4a3). Single cell PCR for AE2 confirmed transcript in post-migratory GnRH neurons.
(TIF)

S2 Fig AE3 is not detected the cell soma or processes of GnRH neurons. A) AE3 (red) was not detected in GnRH neurons (green) in adult mice. B) GnRH fibers (green) in the median eminence of pre-pubertal (PN11; B1) and adult mice (B2) were also negative for AE3 (red), which is highly expressed in the outer region of the median eminence. Scale bars: A = 10 μM, B = 100 μM.
(TIF)
