# Supplementary figures and images for: Chloride Accumulators NKCC1 and AE2 in Mouse GnRH Neurons: Implications for GABAA Mediated Excitation
Source: PLoS One. 2015 Jun 25;10(6):e0131076. doi: 10.1371/journal.pone.0131076 (PMC4482508; doi:10.1371/journal.pone.0131076)

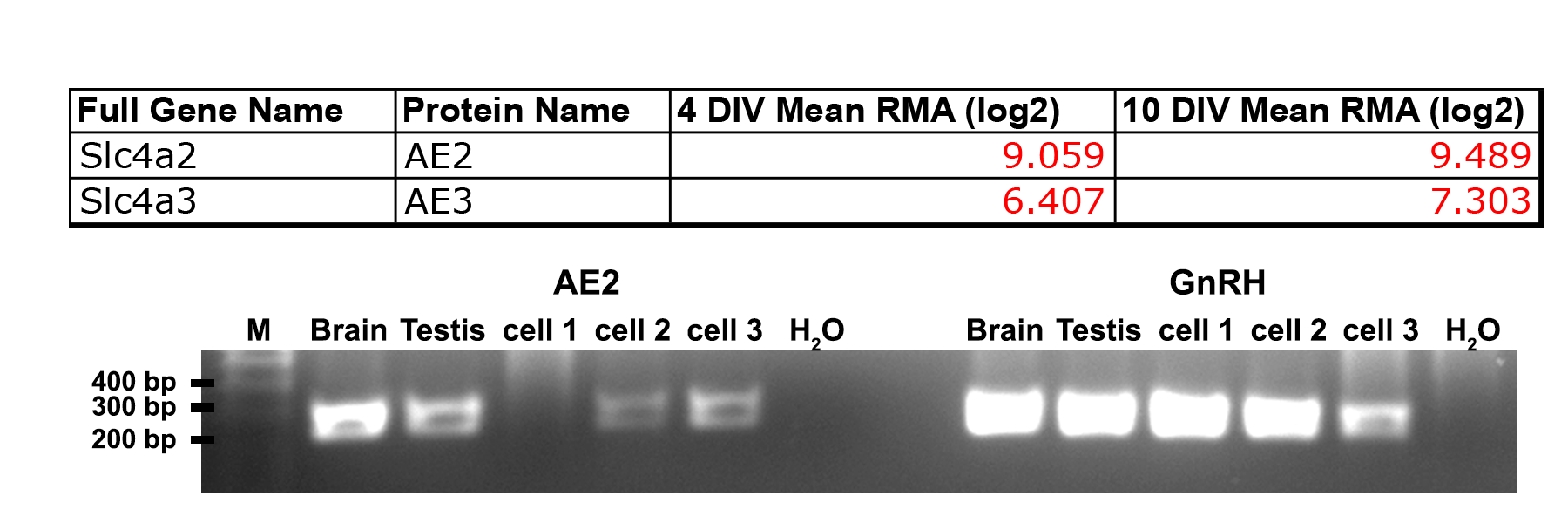

Supplement: S1 Fig — Microarray data generated in our lab (Kramer and Wray 2000) shows Mean mRNA transcript levels for 9 pooled single cell cDNAs in migrating (4 div) and post-migrating (10 div) GnRH neurons for AE2 (Slc4a2) and AE3 (Slc4a3). Single cell PCR for AE2 confirmed transcript in post-migratory GnRH neurons. (TIF) [file pone.0131076.s001.tif]

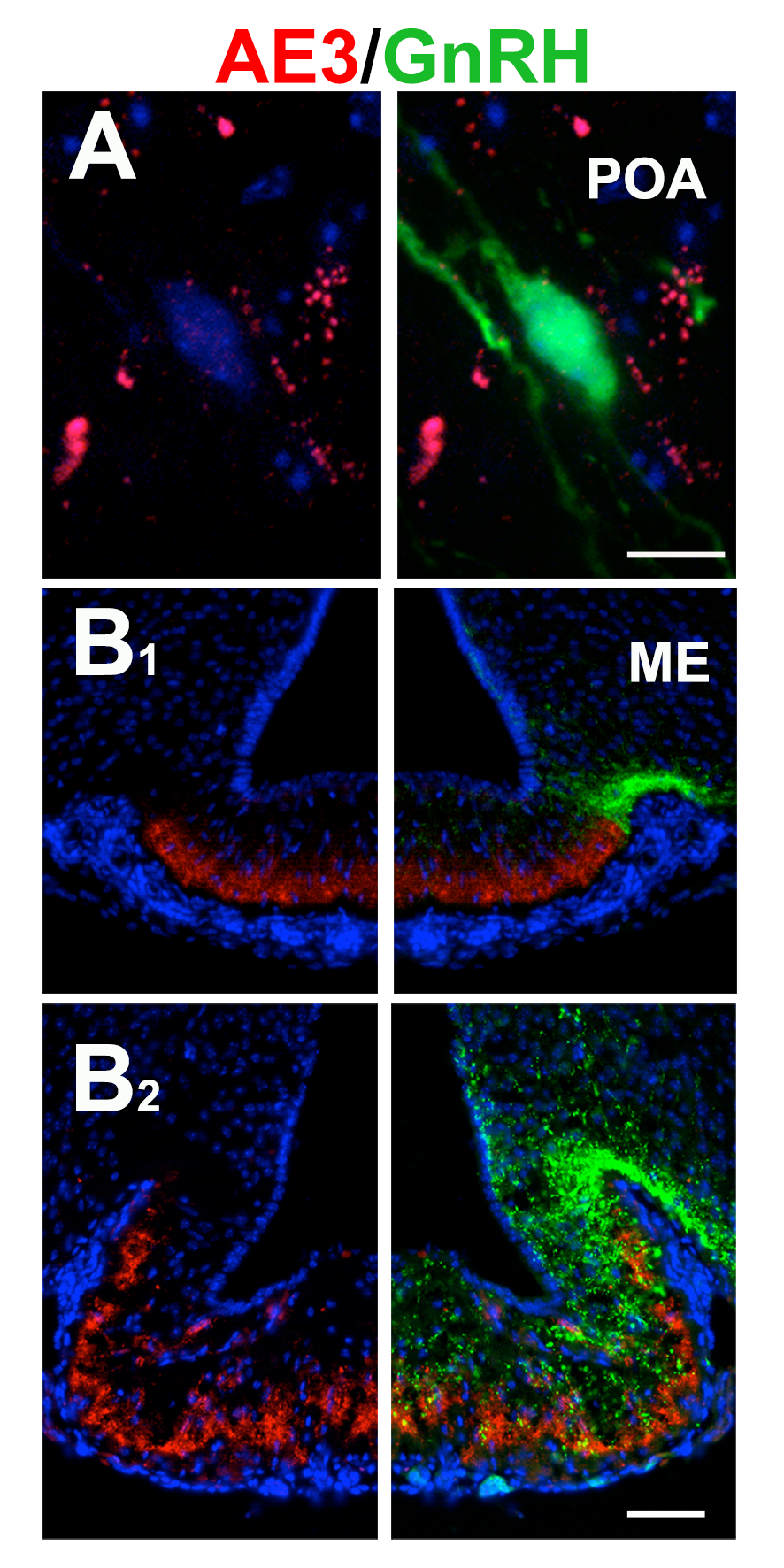

Supplement: S2 Fig — A) AE3 (red) was not detected in GnRH neurons (green) in adult mice. B) GnRH fibers (green) in the median eminence of pre-pubertal (PN11; B1) and adult mice (B2) were also negative for AE3 (red), which is highly expressed in the outer region of the median eminence. Scale bars: A = 10 μM, B = 100 μM. (TIF) [file pone.0131076.s002.tif]
